# Molecular and structural considerations of TF-DNA binding for the generation of biologically meaningful and accurate phylogenetic footprinting analysis: the LysR-type transcriptional regulator family as a study model

**DOI:** 10.1186/s12864-016-3025-3

**Published:** 2016-08-27

**Authors:** Patricia Oliver, Martín Peralta-Gil, María-Luisa Tabche, Enrique Merino

**Affiliations:** 1 Departmento de Microbiología Molecular, Instituto de Biotecnología, Universidad Nacional Autónoma de México, Cuernavaca, Morelos México; 2 Escuela Superior de Apan de la Universidad Autónoma del Estado de Hidalgo, Carretera Apan-Calpulalpan, Km 8, Chimalpa Tlalayote s/n, Colonia Chimalpa, Apan, Hidalgo México

**Keywords:** Phylogenetic footprinting analysis, Motif profile, Transcription factors, Binding sites, Transcription regulation, LysR-type transcription regulator family, LTTR

## Abstract

**Background:**

The goal of most programs developed to find transcription factor binding sites (TFBSs) is the identification of discrete sequence motifs that are significantly over-represented in a given set of sequences where a transcription factor (TF) is expected to bind. These programs assume that the nucleotide conservation of a specific motif is indicative of a selective pressure required for the recognition of a TF for its corresponding TFBS. Despite their extensive use, the accuracies reached with these programs remain low. In many cases, true TFBSs are excluded from the identification process, especially when they correspond to low-affinity but important binding sites of regulatory systems.

**Results:**

We developed a computational protocol based on molecular and structural criteria to perform biologically meaningful and accurate phylogenetic footprinting analyses. Our protocol considers fundamental aspects of the TF-DNA binding process, such as: *i*) the active homodimeric conformations of TFs that impose symmetric structures on the TFBSs, *ii*) the cooperative binding of TFs, *iii*) the effects of the presence or absence of co-inducers, *iv*) the proximity between two TFBSs or one TFBS and a promoter that leads to very long spurious motifs, *v*) the presence of AT-rich sequences not recognized by the TF but that are required for DNA flexibility, and *vi*) the dynamic order in which the different binding events take place to determine a regulatory response (i.e., activation or repression). In our protocol, the abovementioned criteria were used to analyze a profile of consensus motifs generated from canonical *Phylogenetic Footprinting Analyses* using a set of analysis windows of incremental sizes. To evaluate the performance of our protocol, we analyzed six members of the LysR-type TF family in Gammaproteobacteria.

**Conclusions:**

The identification of TFBSs based exclusively on the significance of the over-representation of motifs in a set of sequences might lead to inaccurate results. The consideration of different molecular and structural properties of the regulatory systems benefits the identification of TFBSs and enables the development of elaborate, biologically meaningful and precise regulatory models that offer a more integrated view of the dynamics of the regulatory process of transcription.

**Electronic supplementary material:**

The online version of this article (doi:10.1186/s12864-016-3025-3) contains supplementary material, which is available to authorized users.

## Background

Gene regulation is a key feature of all organisms in response to intracellular and environmental changes. Bacterial gene regulation primarily occurs at the beginning of transcription by transcription factors (TFs) that recognize specific regions near promoter sequences and results in the activation or repression of the transcription of the nearby genes. The number of TFs in prokaryote genomes typically scales as the square of the total number of their genes [1]. For example, for the model organism *Escherichia coli*, with 4,405 genes, approximately 8 % of these genes have been estimated to code for predicted or known TFs [2], of which 35 % correspond to activators and 43 % to repressors, while 22 % have dual activities [3].

The *in silico* identification of transcription factor-binding sites (TFBSs) is a key issue for many molecular biology studies aimed at characterizing regulatory elements in genome sequences. These analyses have been performed by considering either different co-regulated genes in one genome [4] or a set of upstream regions of orthologous genes in closely related genomes, a procedure known as *phylogenetic footprinting analysis* [5–8]. In any case, it is assumed that the nucleotide conservation of a specific region in the set of sequences is indicative of a selective pressure required for the recognition of TFs for their corresponding TFBSs. Based on this principle, the goal of many programs that have been developed to find TFBSs has been the identification of discrete sequence motifs that are significantly over-represented in a given set of sequences where a TF is expected to bind. These motifs are considered to be part of the TFBSs and are commonly represented as position-specific scoring matrices (PSSMs). TFBSs and their corresponding PSSMs have been compiled in a number of different databases, such as RegulonDB [9], EcoCyc [10], RegPrecise [11], Prodoric [12] and Tractor_DB [13]. To evaluate the significance of these TFBS predictions, different approaches have been developed based on theoretical models, such as log-odds, entropy-weighted values [14] or the combination of theoretical and empirical score distributions [15]. Despite their extensive use, the accuracies reached with these programs remain low. In many cases, the true TFBSs are excluded from the identification process or are imprecisely identified, especially when they correspond to low-affinity but important binding sites of the regulatory systems. In other words, the significance of a motif given its over-representation in a set of sequences of co-regulated genes is not necessarily the best way to identify the set of TFBSs for a given regulon.

**Fig. 1:**
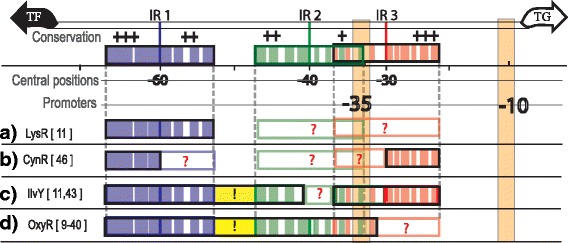
Common types of incorrectly identified regulatory motifs in phylogenetic footprinting analyses that do not correspond to real TFBSs of the LysR-type family in Gammaproteobacteria. The TFs belonging to the LysR-type family in Gammaproteobacteria are commonly transcribed in a divergent orientation with respect to their TGs. In the intergenic region of the TF-TG, there are two to three IRs, represented by purple (IR1), green (IR2) and red (IR3) boxes. The -35 and -10 boxes of the TG are represented by cream rectangles. The question marks represent motif regions that are not commonly identified, while the exclamation marks represent DNA regions that are not part of the regulatory motif but were identified as such. Due to the molecular bases of the regulatory systems, each TFBS was recognized with different affinities by their corresponding TF. Therefore, their sequence conservation varies, wherein IR1 is the most conserved sequence and IR2 is the least conserved sequence. Additionally, the sequence conservation within an IR sequence also presents important differences. Colored spaces within the boxes represent nucleotides of the motif that are more conserved, while white spaces represent poorly conserved nucleotides. Additionally, nucleotide conservation levels of the motifs are represented with plus signs (+), with +++ (three plus signs) indicating DNA regions with the most conserved nucleotides and + (one plus sign) indicating less conserved DNA regions. In each example, the name of the TF of the regulatory system and its corresponding references are indicated. **a** Only IR1, the most conserved of the IRs, is identified. **b** Only the most conserved parts of IR1 and IR3 are identified. **c** A large DNA region including IR1, the most conserved part of the IR2 and IR3 are identified. Additionally, the DNA regions between IR1 and IR2 that are not recognized by the TF are also incorrectly included. **d** A contiguous long DNA region, including the contiguous IR1 and IR2 sequences and the sequence between them, are reported as the TF-binding sequence

Herein, we present a new computational protocol, termed **P**hylogenetic **Pro**file of **Co**nsensus **M**otifs (PProCoM), which is based on the construction of profiles obtained from a set of consensus motifs of canonical *phylogenetic footprinting* techniques, using analysis windows of different incremental sizes. This profile of motifs was further examined considering the fundamental aspects of the TF-DNA binding process, such as: *i*) the active homodimeric conformations of some TFs impose symmetric structures on the DNA binding sites (these symmetric DNA sites can be classified as directed repeat (DR) or inverted repeat (IR) sequences); *ii*) the possibility of the TFs binding cooperatively in adjacent low-affinity sites (these low-affinity sites are commonly not included in the reported TFBS; see Fig. 1a and b); *iii*) the effects of the presence or absence of low-molecular weight effectors, known as co-inducers; *iv*) the incorrect assignment of very long spurious motifs due to the proximity of two or more TFBSs or the proximity of one TFBS to a promoter sequence (see Fig. 1c and d); *v*) the improper inclusion of AT-rich sequences in the reported motifs not recognized by the TF but that are over-represented because they provide the DNA flexibility required for TF binding (see Fig. 1a to d); and *vi*) the dynamic order in which the different binding events take place to determine the appropriate regulatory response (i.e., activation or repression) of the system.

To evaluate the performance of our protocol, we analyzed the regulatory system of six members of the LysR-type transcriptional regulator family in Gammaproteobacteria. This family represents one of the most important families of TFs in bacteria with poorly conserved TFBSs. The members of this family have three domains: the N-terminal domain, which contains a helix-turn-helix motif for DNA binding; a central domain involved in co-inducer recognition; and a C-terminal domain required for both DNA binding and co-inducer response [16]. For most of the cases in our study, we identified TFBSs with different sequence-conservations and, thus, different affinity strengths. In our study, we found that all identified TFBSs were biologically meaningful and allowed us to propose precise dynamic regulatory models.

## Results

To assess the performance of our protocol, we performed *in silico* identifications of the binding sites of TFs of six regulatory systems that are members of the LysR-type family in Gammaproteobacteria, with target genes (TGs) commonly transcribed in divergent orientations. For comparative purposes, we divided these systems into three different groups in accordance with the regulatory activity of the TF on its TG and the position of the TFBSs with respect to the promoter sequences of the regulated genes.

### Group one: GcvA and MetR

**Fig. 2:**
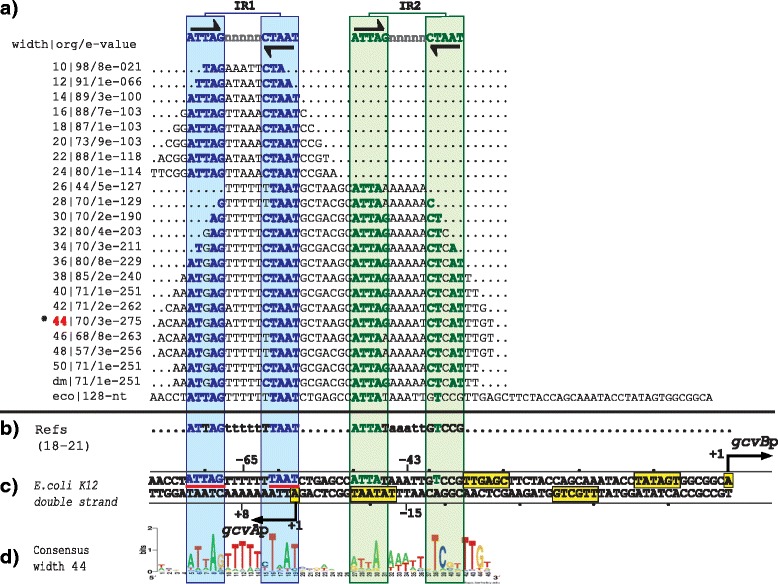
PProCoM analysis of the *gcvA-gcvB* intergenic regions in Gammaproteobacteria. **a** Profile of multiple consensus sequences of increasing length positioned relative to the *E. coli* K12 *gcvA*-*gcvB* intergenic region. In the left column, separated by a pipe, the window width used in each MEME analysis, the E-value obtained for each motif and the number of organisms presenting the identified motif (out of 150 Gammaproteobacteria used in our analysis) are indicated. The last of these consensus sequences is indicated as dm and corresponds to the **d**efault **m**otif without forcing the size of the analysis window (see the Methods section). The consensus motifs of the IR sequences (IR1 and IR2) are displayed at the top of the figure and are represented with inverted black arrows. **b** TFBSs with experimental reported evidence, with references cited on the left side of the figure. **c** Each one of the identified motifs was mapped into the *E. coli* K12 *gcvA*-*gcvB* intergenic region and was used as a reference. Black arrows indicate TSSs that had been previously identified or proposed in our study. The -35 and -10 promoter boxes are indicated with yellow boxes. TSSs and -35 and -10 promoter boxes are indicated with solid lines if these elements had been previously reported and with dashed lines if these elements were identified based on our PProCoM analysis. The center positions of the IR motifs related to the beginnings of transcription of the genes coding for the TF or TG are indicated. The nucleotides of the *E. coli* IR1 sequence, matching the consensus, are underlined with red lines. **d** A LOGO corresponding to a representative consensus was selected from the profile of a consensus of the section (marked with a red asterisk) and is shown. This LOGO includes all of the regulatory motifs of the intergenic region of study

**Fig. 3:**
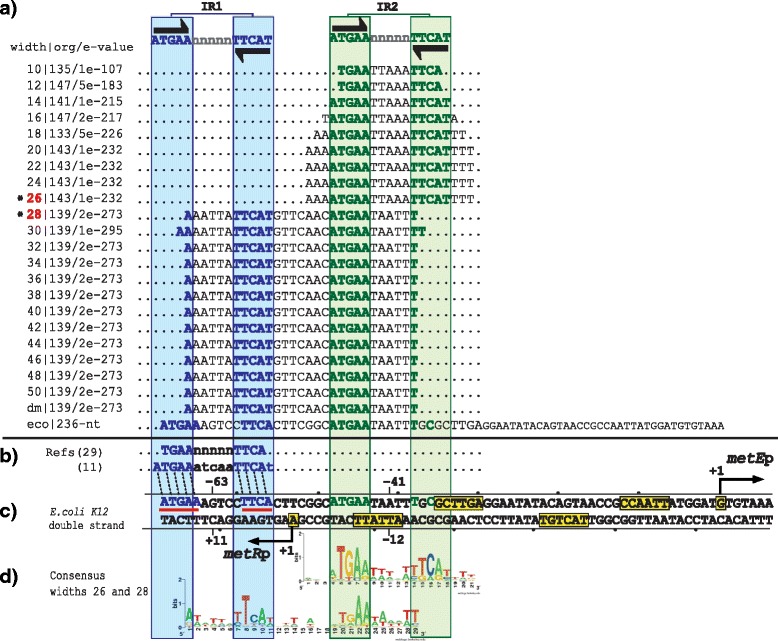
PProCoM analysis of the *metR-metE* intergenic regions in Gammaproteobacteria. The descriptions of sections (**a** to **d**) and the symbols are the same as those of Fig. 2. **d** None of the motifs obtained using the different analysis window sizes include all IR sequences of the intergenic *metR-metE* region; therefore, the LOGOs of two different window sizes are included

The group one is composed of two regulatory systems with the TFs GcvA and MetR. In the intergenic sequences of these regulatory systems, our PProCoM analysis identified two TFBSs (IR1 and IR2, Figs. 2 and 3). The genes coding for the TFs and TGs are transcribed in opposite directions. The transcriptional activation of the TG occurs when one dimer of the TF binds to a TFBS located adjacent the -35 box of the TG promoter and interacts with the RNA polymerase (RNAP; IR2 of Figs. 2 and 3). Simultaneously, the self-repression of the TF occurs when it binds to a TFBS that overlaps its own promoter located on the opposite strand of the DNA (IR1 of Figs. 2 and 3).

### The GcvA regulatory system

GcvA, **G**lycine **C**lea**v**age **A**, is a TF that regulates the transcription of genes involved in the serine-glycine pathway of *E. coli* [17, 18]. This regulator is encoded by the divergent operon *gcvA-gcvB* from overlapping promoters and has a common regulatory mechanism. In the presence of glycine, GcvA is negatively auto-regulated and coordinately increases the transcription of the *gcvB* divergent gene coding for a small RNA [19] by direct interaction with α and β RNAP [20]. Additionally, GcvA regulates the transcription of the *gcvTHP* operon [18].

Using DNase I footprint analysis, Wilson et al. reported that in *E. coli*, GcvA protects a large 48-bp sequence in the intergenic region of *gcvA-gcvB* and two other sequences 35 and 57 bp upstream the *gcvTHP* operon [18]. Alignment of these sequences revealed a conserved 5′-CTAAT-3′ motif, which was subsequently determined by site-directed mutagenesis to be important for GcvA binding and the negative regulation of the *gcvTHP* and *gcvA* transcription units [18, 21, 22]. In general, the GcvA-binding sites do not present a clear sequence conservation, except for a short 5′-CTAAT-3′ motif. Additionally, the protected regions of GcvA contain the IR sequence, 5′-ATTA-n_7_-TAAT-3′ [18], which is coincident with the GcvA-binding site reported in the RegPrecise database [11].

Our PProCoM analysis of the *gcvB-gcvA* intergenic region identified the presence of two 15-bp IR sequences (5′-ATTAG-n_5_-CTAAT-3′, see Fig. 2). These IR sequences include the previously mentioned motifs reported by Wilson et al., 5′-CTAAT-3′ and 5′-ATTA-n_7_-TAAT-3′ [18]. Considering the *E. coli gcvA-gcvB* intergenic region, the central positions of the predicted IR1 and IR2 motifs are located -65 and -43 bp from the *gcvB* transcription start site (TSS), respectively (see Fig. 2). It is important to remark that the sequences shown in this figure are not the result of a standard sequence alignment but are obtained from the relative location of conserved motifs of different sizes to the *E. coli gcvB-gcvA* regulatory region (see the Methods section).

### The MetR regulatory system

MetR is a TF that regulates the expression of genes involved in methionine biosynthesis and protection against nitric oxide [23–28]. The transcriptional activity of MetR is modulated by homocysteine, the metabolic precursor of methionine. In the presence of homocysteine, MetR activates the transcription of some genes, such as *metE* and *glyA*, and represses the transcription of a few others, such as *metH*, *metA*, and *hmp*, along with its own transcription [23–31].

In *E. coli* and *Salmonella typhimurium*, the *metE* and *metR* genes are divergently transcribed from overlapping promoters; thus, they share a common regulatory region [23–29]. DNase I footprint and mutational analyses in *S. typhimurium* showed that MetR binds to two IR sequences arranged in tandem with different affinities to consensus sequence 5′-TGAAnnTnnTTCA-3′ [29]. In *E. coli*, two binding sites with the same characteristics have been reported in the regulatory region of the divergently transcribed *hmp-glyA* genes regulated by MetR, [30, 31]. The presence of homocysteine has been postulated to enhance the affinity of MetR to these contiguous DNA-binding sites to activate *metE* and repress *metR* transcription [29]. To date, no experimental evidence supports the existence of two MetR-binding sites on the *E. coli metR-metE* intergenic region. Nevertheless, based on our PProCoM analysis, we identified two 15-bp IR sequences with consensus sequence 5′-ATGAA-n_5_-TTCAT-3′, which is the reported size of the TFBSs of the LysR-type TF family [16]. Based on the *E. coli* reference sequence, we localized the distal and more conserved IR1 site -63 bp from the *metE* TSS, while the proximal and less conserved site, IR2, was located -41 bp from the TSS of *metE* (Fig. 3). These central locations are among the preferred positions of the transcriptional activators in *E. coli* [32, 33]. As shown in Fig. 3, the *E. coli* IR1-IR2 inter-motifs sequence is one base shorter than the IR1-IR2 inter-motifs sequence of the overall PProCoM motif alignment (see Fig. 3, sloped-dotted lines). The effect, if any, of this one missing base in the *E. coli metR-met*E inter-motifs space on the system regulation is not clear. Nevertheless, longer variations in the inter-motifs space, such as 6 bases (half-helix turn), have been demonstrated to have a negative effect on *S. typhimurium metE* transcription [34]. Additionally, point mutations in any of the two proposed TFBSs have also been reported to decrease the *metE* transcription, indicating that both TFBSs are required for full *metE* activation [29]. The 15-bp consensus sequence obtained from our PProCoM analysis was coincident with that reported for MetR in the RegPrecise database, i.e., 5′-ATGAAAATTTTTCAT-3′ [11].

### Group two: OxyR, IlvY and CynR

**Fig. 4:**
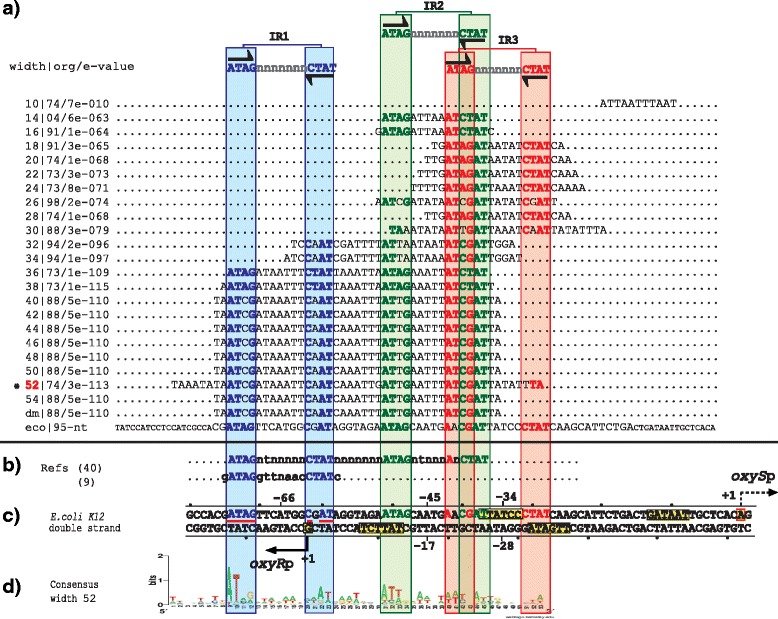
PProCoM analysis of the *oxyR-*oxyS intergenic regions in Gammaproteobacteria. The descriptions of sections (**a** to **d**) and the symbols are the same as those of Fig. 2

**Fig. 5:**
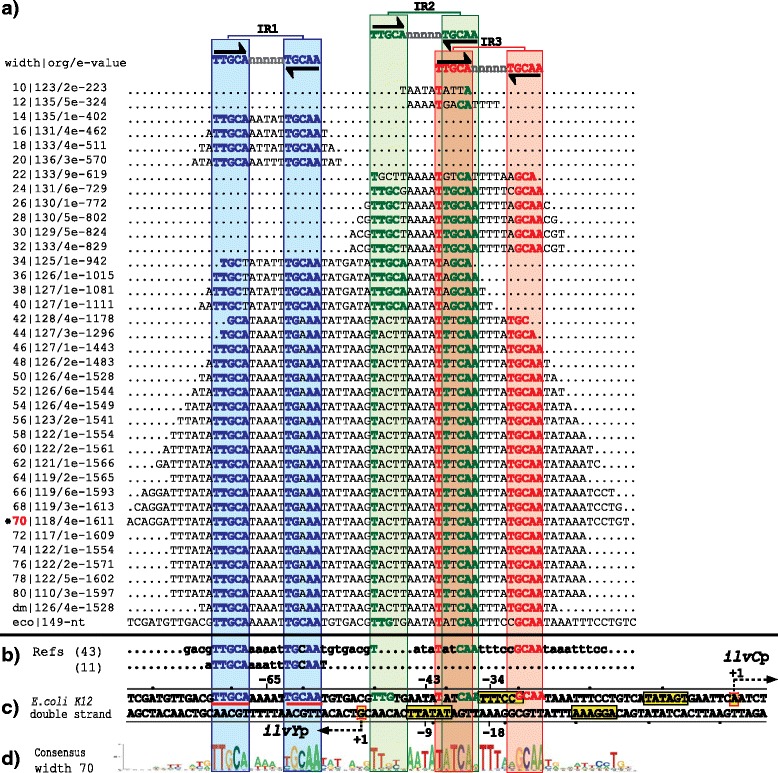
PProCoM analysis of the *ilvY-ilvC* intergenic regions in Gammaproteobacteria. The descriptions of sections (**a** to **d**) and the symbols are the same as those of Fig. 2

**Fig. 6:**
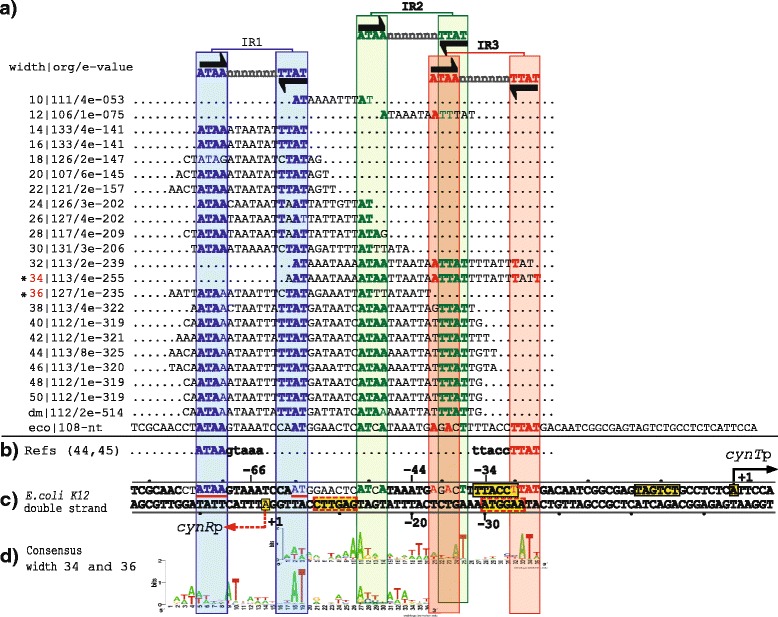
PProCoM analysis of the *cynR-cynT* intergenic regions in Gammaproteobacteria. The descriptions of sections (**a** to **d**) and the symbols are the same as those of Fig. 2. **d** None of the motifs obtained using the different analysis window sizes includes all IR sequences of the intergenic *cynR-cynT* region; therefore, the LOGOs of two different window sizes are included

Group two is composed of three regulatory systems with the TFs OxyR, IlvY and CynR. Our PProCoM analyses of these regulatory systems identified three TFBSs in their corresponding intergenic regulatory regions (IR1, IR2 and IR3; Figs. 4, 5 and 6). The transcriptional activation of the TG occurs by the cooperative binding of two dimers that recognize IR1 and IR2 in the presence of their respective inducers. Because IR2 is located adjacent to the -35 box of the TG promoter, the TF dimer bound to this site promotes TG transcription by its interaction with the RNAP. Additionally, the self-repression of the TF simultaneously occurs because IR1 overlaps the TF promoter located on the opposite strand of the DNA (IR1 of Figs. 4, 5 and 6). The main difference with respect to our group one of regulatory systems is the presence of a third TFBS that overlaps the -35 box of the TG promoter (IR3; Figs. 4, 5 and 6). A remarkable characteristic of this group is that this third TFBS (IR3, used for TG repression) partially overlaps the second TFBS (IR2, used for TG activation) in such a way that the binding of the TF to one of these two mutually exclusive sites determines the transcriptional regulatory activity (i.e., activation or repression) on the TF over the TG.

### The OxyR regulatory system

OxyR is a TF that regulates the expression of genes involved in oxidative stress protection, redox balance, and manganese uptake [35–38]. The transcriptional activity of OxyR depends on its oxidized state, which determines the reversible disulfide bond formation of a pair of cysteine residues in its amino acid sequence [39]. In its oxidized state, OxyR activates the transcription of the divergent small RNA gene *oxyS*. Additionally, OxyR represses its own expression under oxidizing and reducing conditions [40].

Based on DNase I footprint analyses, Tartaglia et al. showed that OxyR binds to an unusually long DNA region that spans over 45 bp, with putative OxyR-binding sites with no obvious sequence similarity [41]. Using an in vitro binding assay of OxyR to random oligonucleotides and DNase I footprint analyses, Toledano et al. showed that the DNA recognition of OxyR depends on its oxidized/reduced states. In its oxidized form, OxyR recognizes a DNA region that includes four repetitions of the 5′-ATAGnt-3′ sequences located in four contiguous major grooves on one face of the DNA helix. In its reduced form, OxyR binds two repetitions of the 5′-ATAGnt-3′ sequences located at two pairs of major grooves separated by one helical turn [40].

Our PProCoM analysis of the *oxyR-oxyS* intergenic region identified the presence of three 15-bp IR sequences (5′-ATAG-n_7_-CTAT-3′). Considering the *E. coli oxyR-oxyS* intergenic region, the central positions of the predicted IR1, IR2 and IR3 motifs are located -66, -44 and -35 bp from the *oxyS* TSS, respectively (see Fig. 4).

### The IlvY regulatory system

IlvY positively regulates the transcription of *ilvC*, a gene involved in isoleucine and valine biosynthesis. The transcriptional activation of *ilvC* by IlvY depends on the presence of an IlvY inducer, such as acetolactate or acetohydroxybutyrate. At the same time, IlvY negatively regulates its own transcription in an inducer-independent manner [42, 43].

The *ilvY* and *ilvC* genes are divergently transcribed from overlapping promoters. Using DNase I footprint analyses, Wek and Hatfield proposed that IlvY binds to two 27-bp operator sequences, named O1 and O2, in the *ilvY*-*ilvC* intergenic region [43]. These regions are arranged in tandem and possess imperfect 21-bp inverted repeat motifs: O1, 5′-ACgTTGCAAaaaTTGCAAtGT-3′ (centered at position +17 relative to the *ilvY* TSS), and O2, 5′-aTATatCaatttccGcaATAa-3′ (which overlaps the proposed -10 and -35 promoter boxes of *ilvY* and the -35 promoter box of *ilvC*). The consensus IlvY-binding motif common to the O1 and O2 operators is 5′-A[C/T]ATTGCAA-3′ [43]. These authors proposed that IlvY represses its own transcription in an inducer-independent manner when IlvY binds to O1 and activates transcription of *ilvC* when IlvY binds to the O1 and O2 operators in a cooperative dependent manner in the presence of the system inducers. In this condition, the transcriptional activation of *ilvC* was proposed to result from IlvY-RNAP interactions when IlvY was bound to O2 or by a change in the DNA conformation at the *ilvC* -35 promoter box. Following this reasoning, Rhee et al. proposed that the transcription of the divergent genes *ilvY* and *ilvC* is coupled in a DNA supercoiling-dependent manner that increases the binding of the RNAP at this promoter by nearly 100-fold [42].

Our PProCoM analysis of the *ilvY-ilvC* intergenic region identified the presence of three 15-bp IR sequences (5′-TTGCA-n_5_-TGCAA-3′; see Fig. 5). Considering the *E. coli ilvY-ilvC* intergenic region, the central positions of these predicted IR1, IR2 and IR3 motifs are located -65, -43 and -34 bp from the *ilvC* TSS, respectively (see Fig. 5).

### The CynR regulatory system

CynR is a TF that regulates the transcription of the *cynTSX* operon, which is involved in cyanate detoxification. Cyanate is also used as a nitrogen source due to its hydrolysis to ammonia and bicarbonate [44]. This activation of the *cynTSX* operon by CynR depends on the presence of cyanate. CynR also negatively regulates its own transcription in a cyanate-independent manner [44].

As in the case of the abovementioned LysR-type regulatory systems, the gene coding for the TF (*cynR*) and its regulatory TGs (*cynTSX*) are transcribed in opposite directions, and their corresponding promoters overlap [45, 46]. Using DNase I digestion analyses, Lamblin and Fuchs showed that CynR binds to a 60-bp region in the *cynR*-*cynTSX* intergenic region and proposed that this region contains two putative binding sites with different affinities [46]. The first of these regions, R1 (5′-ATAAGTAAA-3′), was proposed to have the highest binding affinity, whereas the second region, R2 (5′-ATAAGGTAA-3′), was proposed to overlap the entire *cynR* promoter sequence and the -35 promoter region of the *cynTSX* operon [45, 46]. These authors suggested that in a first instance, a CynR dimer could bind to R1 (i.e., the most conserved region), and in a second but almost simultaneous instance, another CynR dimer could bind to R2 in a strong cooperative manner. These authors also proposed that the transcriptional activation of the *cynTSX* operon takes place in the presence of cyanate, which was believed to trigger a conformational change in CynR, modifying its interaction with DNA [46].

Our PProCoM analysis of the *cynR-cynTSX* intergenic region identified the presence of three 15-bp IR sequences (5′-ATAA-n_7_-TTAT-3′), including the sequences proposed by Lamblin and Fuchs (see Fig. 6). Considering the *E. coli cynR-cynTSX* intergenic region, the central positions of the predicted IR1, IR2 and IR3 motifs are located -66, -44 and -34 bp from the *cynTSX* TSS, respectively (see Fig. 6).

### Group three: LysR

**Fig. 7:**
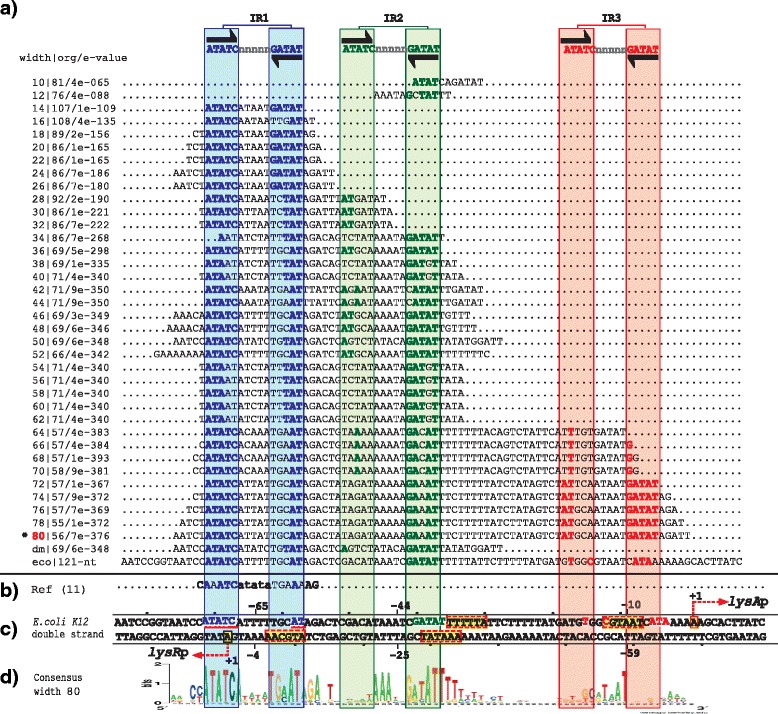
PProCoM analysis of the *lysR-lysA* intergenic regions in Gammaproteobacteria. The descriptions of sections (**a** to **d**) and the symbols are the same as those of Fig. 2

The group three is composed of one regulatory system with the TF, LysR. Our PProCoM analysis of this regulatory system identified three TFBSs in the *lysR-lysA* intergenic regulatory region (IR1, IR2 and IR3; Fig. 7). As observed in the groups one and two of the LysR-Type family, the transcriptional activation of the TG (*lysA*) occurs by the cooperative binding of two dimers, which recognize the IR1 and IR2 sites in the presence of a LysR inducer (i.e., diaminopimelic acid). The self-repression of the TF (*lysR*) occurs simultaneously because IR1 overlaps the TF promoter located on the opposite strand of the DNA (IR1 of Fig. 7). The main difference with respect to the regulatory system of the group two is that the second and third TFBSs do not overlap (IR2 and IR3; Fig. 7).

### The LysR regulatory system

LysR is a TF that regulates the transcription of *lysA*, which encodes an enzyme that catalyzes the final step of lysine biosynthesis. LysR negatively regulates its own transcription and positively regulates the transcription of *lysA* in the presence of its inducer, diaminopimelic acid [47–49].

As in the previous cases, the genes coding for the TF (*lysR*) and its regulatory TG (*lysA*) are transcribed in opposite directions. The TFBSs of LysR and their regulatory mechanism have not yet been identified. However, the LysR-binding sites have been determined to be within a 73-bp DNA fragment located 48 bp upstream of the *lysA* structural gene [48]. The intracellular concentration of active LysR could be limiting because its regulatory role is diminished when the abovementioned fragment is cloned on plasmids [48]. Based on experimental analyses, the *lysR* TSS has been predicted to be located 26 bp upstream of its structural gene [9]. However, a putative *lysA* promoter, with a -35 box (TTGcat) and a -10 box (TATTTT), has been predicted to be located 52 bp from the *lysA* coding region [50]. The corresponding TSS has been proposed to be located 3 bp downstream of the -10 box of the predicted promoter [50].

Our PProCoM analysis of the *lysR-lysA* intergenic region identified the presence of three 15-bp IR sequences (5′-ATATC-n_5_-GATAT-3′, see Fig. 7). Considering the *E. coli lysR-lysA* intergenic region, the central positions of the predicted IR1, IR2 and IR3 motifs are located -64, -43 and -9 bp from the *lysA* TSS, respectively (see Fig. 7). Based on the positions of these predicted TFBSs, we postulate that the *lysA* TSS is located 22 bp upstream of its structural gene.

## Discussion

### Common sequence motifs of the TFBSs of the LysR-type TF family

**Fig. 8:**
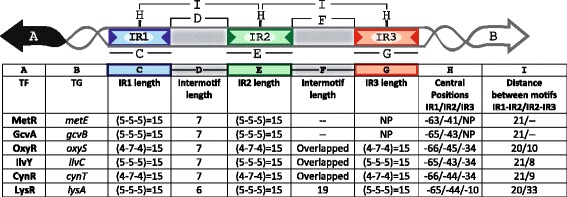
Architecture of the TFBSs of the LysR-type TF family in Gammaproteobacteria revealed by PProCoM analysis. A common characteristic of the members of the LysR-type family in Gammaproteobacteria is that their coding genes and corresponding target genes are transcribed in divergent orientations, and their intergenic regions present two or three inverted repeated motifs, IR1, IR2 and IR3. The architecture of the intergenic regions of the six TFs analyzed in our study is summarized. Clear conservations of motif length and inter-motif distance suggest that there are similarities in their molecular regulatory mechanisms

**Fig. 9:**
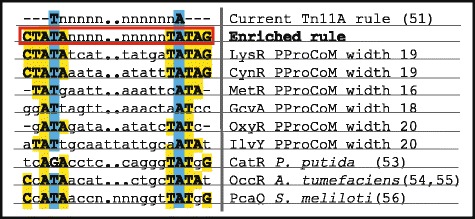
Consensus sequence for the TFBSs of the LysR-type TF family. The T-n_11_-A motif was originally proposed by Goethals et al. [51] as the consensus sequence recognized by members of the LysR-type family. Considering the results of our PProCoM analysis of the TFBSs of six representative members of this family in Gammaproteobacteria, we defined a new and extended version of this motif: 5′-CTATA-n_9_-TATAG-3′. Additionally, examples of the sequence consensus of the TFBSs of other members of the LysR-type family that have been experimentally verified are also shown and include the distal TFBSs of CatR of the Gammaproteobacteria *Pseudomonas putida* [53], OccR of the Alphaproteobacteria *Agrobacterium tumefaciens* [53, 54] and PcaQ of the Alphaproteobacteria *Sinorhizobium meliloti* [56]. Dots within the inter-motif sequences were used to align the conserved nucleotides of the consensus sequences

Our systematic analyses of the six representative members of the LysR-Type family in Gammaproteobacteria using our PProCoM protocol allowed us to precisely identify their corresponding TFBSs and common characteristics, which are summarized in Fig. 8. The genes coding for the TFs and their TGs are transcribed in divergent orientations. Their intergenic regions present two or three inverted repeated motifs, i.e., IR1, IR2 and IR3. Based on this figure, the IR sequences are clearly conserved in terms of their length and common inter-motif distances and also show similarities in their molecular regulatory mechanisms. In addition to these specific sequence conservations, the correct identification of the TFBSs of these LysR-type TFs enables us to identify conserved sequence similarities represented by the consensus sequence 5′-CTATA-n_9_-TATAG-3′, as shown in Fig. 9. This consensus sequence can be considered an extended version of the T-n_11_-A “LysR motif”, originally postulated by Goethals et al. and based on the analyses of the TFBSs of NodD in *Azorhizobium* and other members of the LysR-type family [51]. This relevant sequence conservation of the TFBS among members of the LysR-type family can be explained when considering that new genomes frequently acquire these TFs via horizontal gene transfer. Furthermore, these TFs evolved from a common ancestor, as evidenced by the conservation of similar sequences for the binding motifs and the similar molecular mechanisms that regulate the transcriptional responses to a variety of stimuli and functions, including metabolism, quorum sensing, motility and virulence, among others (reviewed in [52]). From Fig. 9, it can also be seen that the most common interspace size between the 5′-CTATA-3′ and 5′-TATAG-3′ monomer recognition motifs is 9 nucleotides. Minor variations of this length can be found; the largest variation was observed for IlvY, with an interspace of 11 nucleotides. Our PProCoM analyses show that this inter-monomer motif sequence has a high AT content to provide the DNA the flexibility required for proper TF-DNA recognition. A few variations to this 5′-CTATA-n_9_-TATAG-3′ consensus exist because they are required for the specific recognition of a TF by its corresponding TFBSs (see Figs. 2, 3, 4, 5, 6 and 7). Figure 9 also includes representative examples of other members of the LysR-type with experimentally characterized TFBSs. These TFBSs are consistent with our extended LysR motif consensus. For example, in the *catR*-*catBC* intergenic region of *Pseudomonas putida*, the distal TFBS of *catBC*, also known as the repressor-binding site, has an imperfect palindromic sequence 5′-tcAgA-n_9_-TATgG-3′ (note the underlined g nucleotide) that resembles our extended LysR motif, 5′-CTATA-n_9_-TATAG-3′. Site-directed mutagenesis of G➔T in the fourth nucleotide of this motif created a sequence most similar to the consensus and resulted in increased binding of the CatR and increased the transcription level of the *catBC* operon. However, substitutions of the first A➔T in the same 5′-tcAgA-n_9_-TATgG-3′ TFBS (note the underlined A nucleotide) made this sequence less similar to the consensus and resulted in decreased binding of CatR, with concomitantly decreased transcription of the *catBC* operon [53]. Our second example corresponds to the OccR regulatory system in *Agrobacterium tumefaciens*. By cloning discrete regions of the *occR*-*occQ* intergenic region and characterizing them using DNase I footprinting and gel mobility shift assays, Wang et al. defined five binding sites of OccR and their relative affinities [54, 55]. Sites 1 and 2 formed an IR located -33 bp from the *occQ* TSS and corresponded to the IR3 site of the IRs used in this work. Sites 4 and 5 form another IR located -64 bp from the *occQ* TSS, corresponding to IR1, the site with the greatest affinity for OccR. Sites 3 and 2 form an IR that corresponds with IR2, i.e., the site with the least affinity of the three IRs of the system [54]. The replacement of IR3 with IR1, i.e., the IR with the greatest affinity, resulted in an enhanced binding of OccR and a greater transcription repression of the *occQ* TG [55]. OccR only binds IR2, i.e., the IR with the smaller affinity, in a cooperative dependent manner in the presence of the system inducer, octopine [55]. Nevertheless, the replacement of IR2 with IR1, i.e., the IR with the greatest affinity, resulted in a partial octopine-independent binding of OccR to this site [55]. Finally, our third example corresponds to the *pcaQ*-*pcaMNVWX* intergenic region in *Sinorhizobium meliloti*. Based on site-directed mutagenesis, McLean et al. proposed that the PcaQ-binding site corresponded to the sequence 5′-ATAaccgggggatTAT-3′ which central position is located 65.5 bp upstream of the structural gene (see Fig. 9). Relevant changes in the nucleotides for TF recognition in our 5′-CTATA-n_9_-TATAG-3′ consensus resulted in decreased transcriptional activation of the target *pcaMNVWX* operon in the presence of its inducer [56]. These mutations involved the A➔G changes at the underlined nucleotides of the sequence 5′-ATA-n_10_-TAT-3′, generating the sequences 5′-GTA-n_10_-TAT-3′, 5′-ATG- n_10_-TAT-3′ and 5′-ATA- n_10_-TGT-3′ [56].

### Dynamic models of regulation

In addition to a static description of the TFBSs identified by our PProCoM analyses, dynamic models of regulation for each one of our systems can be elucidated based on the characteristics of the elements of the regulatory system, which are as follows:

1. The intergenic sequences of the regulatory systems of group one (*metR-metE* and *gcvA-gcvB*), contained two IR motifs, whilst the regulatory systems of group two (*oxyR-oxyS*, *ilvY-ilvC*, and *cynR-cynT*) and group three (*lysR-lysA*) contained three IR motifs. In all these cases, the IR motifs show different sequence conservation, and thus, different affinity. In group one, IR1 is the most conserved, and IR2 is the least conserved motif. In groups two and three, IR1 and IR3 are the most conserved, and IR2 is the least conserved motif.
2. All the TFs analyzed; GcvA, MetR, OxyR, IlvY, CynR and LysR, adopt two different conformations depending on the presence or absence of their corresponding inducers: glycine, homocysteine, reactive oxygen species, acetolactate, cyanate and diaminopimelic acid, respectively.
3. Without the system inducers, the TFs bind as dimers, preferentially to IR1, in the case of group one, and to IR1 and IR3, in the case of groups two and three. In accordance with this binding, footprinting assays with LysR family members show a hypersensitive region 50 bp upstream of the TSS of IlvY [42, 43], CynR [46], OccR [54, 57]. Similar results have been observed in studies with other regulatory TFs of the LysR family such as ClcR [58], CatR [58] and PcaQ [59]. In the case of CynR, this hypersensitive region corresponds to the region where the DNA curves with the binding of CynR [46].
4. In the presence of the system inducers, the TFs bind DNA as dimers of dimers in a cooperative manner. Only through this cooperative binding the TFs can recognize IR2, the less conserved of the TFBSs. This kind of binding for members of the LysR TF family has been demonstrated by footprinting assays [18, 29, 34, 40, 42, 43, 54, 57] and site directed mutagenesis analysis [21, 29, 34, 40, 53–56]. As a consequence of this binding, the hypersensitive DNA regions located around -50 bp upstream the TSSs markedly decrease. In addition, it has been shown that altering the distance between IR1 and IR2 reduces the cooperative binding of the TFs [40, 54, 57].
5. A TFs acts as transcription repressor or activator of the TF or TG genes depending on the position of the IR to which it binds.
6. The IR1 motifs are downstream or overlap the -10 box of the TF promoters, therefore, the auto-repression of transcription takes place when the TFs are bound to IR1 sites.
7. The IR2 motifs overlap the TFs promoters and are also immediately downstream of the -35 box of the TGs promoters, therefore, a TF bound to IR2 represses the TF transcription and activates the TG transcription.
8. In the case of group two, the IR3 motif overlaps the TF and TG promoters, hence, a TF bound to this site simultaneously blocks the transcription of the TF and TG genes. In the case of group three, the IR3 motif only overlaps the TG promoter, accordingly, a TF bound to this site exclusively blocks TF transcription.
9. In addition to the above-mentioned regulatory outcomes, it is worth mentioning that in the case of group two, the IR2 and IR3 sites overlap, therefore, the binding of TFs to these sites are mutually exclusive. In the absence of the system inducers, the TFs would preferentially bind IR3 since this site has greater sequence conservation than IR2; nevertheless, in the presence of the system inducers, the TFs would bind cooperatively as a dimer of dimers to IR1 and IR2. In this case, the binding of the TFs to IR2 would have two positive effects on TG transcription; directly by its interaction with the RNA polymerase, and indirectly, by blocking the binding of the TFs to IR3, an event that otherwise would repress TF transcription.

### Representative regulatory models of the LysR-type TF family in Gammaproteobacteria revealed by PProCoM analyses

**Fig. 10:**
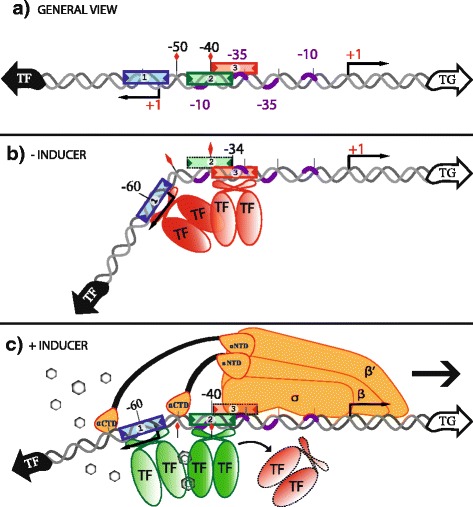
Representative regulatory models of the LysR-type TF family in Gammaproteobacteria revealed by PProCoM analyses. **a** A typical architecture of the regulatory regions of these TFs is the presence of three IR sequences, represented by blue (IR1), green (IR2) and red (IR3) boxes. Some regulatory systems, such as those of our first analysis group, GcvA and MetR, lack the third IR element. **b** Because the sequence affinities of IR1 and IR3 (observed as sequence conservation of the motifs) are greater than the one for IR2, in the absence of the inducer, the TF of the system only binds to the IR1 and IR3 sites. The positions of IR1 and IR3 are critical for the transcriptional repression of divergent systems. IR1 overlaps the TF promoter, while IR3 overlaps the TF and the TG promoters. **c** In the presence of the system inducer, the TF dimer can bind cooperatively to a less conserved and less affine IR in the system, i.e., IR2. A remarkable characteristic of several regulatory systems in this family is that IR2 partially overlaps IR3; therefore, a first consequence of the binding of the TF to IR2 is the steric displacement of the TF that was bound to IR3, resulting in TG transcription repression. In addition to this de-repression effect, a second effect of the binding of the TF to IR2 is direct transcriptional activation of the TG due to the position of IR2 immediately upstream of the -35 promoter box of the TG, where the TF interacts with the RNAP. Figure modified from [57]

In addition to the sequence motifs identified in the TFBSs of the members of the LysR-type TF family, our regulatory model also includes the effects of TF binding on the DNA curvature. These effects have also been reported for several TFs, such as GcvA [18], MetR [30], OxyR [40], IlvY [42], CynR [46], LysR [52], CysB [60], CatR [53], ClcR [58], and OccR [57]. In the above-mentioned regulatory systems, based on DNase footprinting analyses, it has been reported that in the absence of system inducers, TFs bind to long regions of DNA. Conversely, in the presence of inducers, the protected area of the DNA in the footprinting analyses significantly decreases. For example, in absence of the inducer, OccR protects a region of approximately 60 bp, resulting in DNA with a curvature angle of 62°, showing hypersensitive regions around the -50 position [61]. In the presence of the inducer, the angle decreases to 46°, shortening the length of protected DNA in the DNase footprinting assay to 50 bp, decreases the hypersensitive region [57, 61]. Toledano et al. proposed that this reduction in the length of protected DNA was caused by the rearrangement of the dimers of dimers of the TFs. In the absence of inducers, dimers bind to distal sites, e.g., IR1 and IR3. A single turn of the separation between the two dimers causes a bend in the DNA and, consequently, the inhibition of the transcription of divergent transcription units [40]. A similar regulatory model was proposed by Wang and Winans in the *occR*-*occQ* regulatory system [57]. The results obtained with our PProCoM protocol, summarized as our 5′-CTATA-n_9_-TATAG-3′ extended LysR-type TFBS motif consensus (Fig. 9) and schematized in our model (Fig. 10), are consistent with the observations on DNA curvature available in the literature.

### Potential use of PProCoM to identify TFBSs of other regulatory systems different to those of the LysR-Family

Our PProCoM protocol can be used to identify TFBSs of almost any bacterial regulatory system if the characteristics of their TFs are considered. For example, in addition to of the LysR regulatory system, we currently conduct a study to identify the binding sites of the TF members of the AraC/XylS family [62]. These TFs usually bind DNA as dimers to repeated direct asymmetric contiguous TFBSs, being the distal one the most conserved site and proximal site the less conserved. The problem in identifying TFBS of the AraC/XylS family is the low conservation and asymmetry of these proximal TFBSs. Nevertheless, we believe that PProCoM is particularly useful identifying such low conserved binding sites since its accuracy does not exclusively depend on the sequence conservation of the TFBSs, but on the molecular properties of the TFs and their interactions between themselves, with the DNA and with the DNA polymerase. Regarding the use our PProCoM protocol for identifying TFBSs in eukaryotic organisms with small intergenic regions, such as yeast, we consider the possibility of obtaining positive results as obtained so far in prokaryotic organisms. Currently we perform site directed mutagenesis and transcriptional quantification of our regulatory systems for experimental verification of our theoretical predictions.

## Conclusions

PProCoM represents an unconventional multiple motif alignment representing a set of consensus sequences of increasing length, which are arranged according to reference nucleotide intergenic region – *E. coli* sequences in our examples (Figs. 2, 3, 4, 5, 6 and 7). This strategy enables the merging of the represented motifs (with significant E-values) with less conserved motifs that play important roles in dynamic transcription regulation systems. These less conserved motifs have generally not been identified or included in previous studies, even in cases with experimental analyses, such as DNase footprinting analysis. Our PProCoM analysis of six members of the LysR-type TF family have made evident the high relevance of the less conserved motifs in the intergenic regions of their regulatory sequences. This approach enables the comprehension of the homodimeric nature of these TFs and provides a more integrated and complete picture of their regulatory processes.

## Methods

**Fig. 11:**
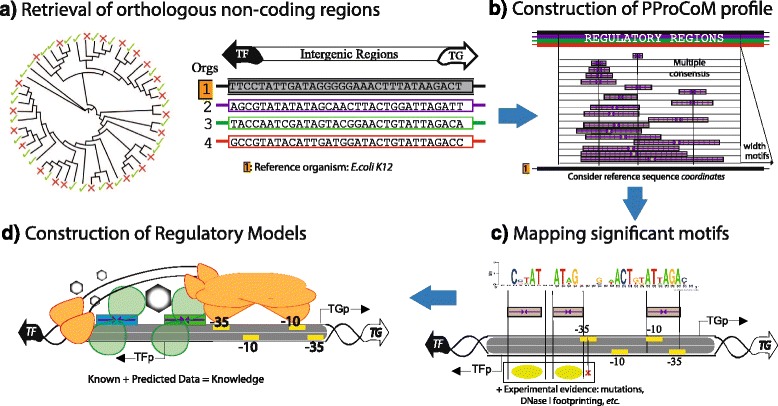
PProCoM workflow. The PProCoM workflow includes four main steps, represented by the units (**a** to **d**). These steps are fully described in the Methods section

In general, our computational PProCoM protocol is an extension of *phylogenetic footprinting analysis* [5]. Briefly, different consensus motifs are obtained using analysis windows with different incremental sizes that are then aligned to build a profile of consensus motifs. This use of windows of different sizes allows for the identification of the most represented (i.e., most significant) sequence motifs and includes other less represented (i.e., less significant) motifs that are nevertheless of equal importance. The fundamental computational steps of our approach are illustrated in Fig. 11 and are described as follows.

### Retrieval of orthologous non-coding regulatory sequences of non-redundant organisms

To avoid bias introduced by the sequencing of preferential model organisms, non-redundant genomes were selected from the KEGG database (release 2015) based on their phylogenetic distances, which were evaluated using the PROTDIST program [63] from a multiple alignment of concatenated sequences of a set of 31 “house-keeping” proteins defined by Ciccarelli et al. [64] (see Fig. 11). The phylogenetic group considered in our study was Gammaproteobacteria. Orthologous genes were defined using “bidirectional best hits” criteria [65] in BLAST [66]. Only intergenic regions with greater length to 10 nucleotides were considered. 150 intergenic regions were considered for analysis. The list of these organisms is presented in Additional file 1: Table S1.

### Obtaining the profile of significantly over-represented motifs from *phylogenetic footprinting* analysis

Over-represented motifs in each orthologous set of regulatory regions were obtained using the Multiple EM for Motif Elicitation (MEME) program [67] considering the following parameter values:

- Length of the analysis window. Although MEME can automatically set the size of the analysis window to define the value at which over-representation of a motif is most significant, in our PProCoM protocol, the MEME analysis was repeatedly performed using analysis windows of different sizes, from the smallest, 10 bp, to the largest, 100 bp, in increments of 2 bp per cycle, or in the case that the results of the analysis remains unchanged despite the increment of the two pair bases. The sizes of the analysis windows were defined using the –*w* argument of MEME. In addition, we also include the result of a MEME analysis without forcing the size of the analysis window. In Figs. 2, 3,4, 5, 6 and 7, these motifs are indicated as dm (**d**efault **m**otif without forcing the size of the analysis window).
- E-value of the motifs. Unlike most computational methods that use the E-value to define a motif as significant, in our PProCoM protocol, the E-value is considered as one, among other different criteria, for the selection of significant motifs. The above consideration is because the E-value of a motif might vary depending on the affinity of the TFBSs (high or low), the size of the analysis window, the number of sequences analyzed and on the phylogenetic distances between the organisms in the study. Nevertheless, as a first filter to define a motif as significant, the E-value was set to 1e-6 using the –*evt* argument of MEME. Figures 2, 3, 4, 5, 6 and 7 include the E-values obtained for each of the analysis window of our six regulatory systems. In all these cases, the E-values were statistically significant (*E*-values < 1e-20).
- Number of motifs identified. To build the PProCoM profile, only the most significant motif is considered per analysis window. This was specified setting the –*nmotifs* argument of MEME to 1.
- Motif symmetry. Considering that some homodimeric TFs, as those of the LysR-family, recognize palindromic DNA sequences, the –*pal* argument of MEME was used to force this symmetry in the identified motifs.
- Distribution of motifs. To specify that the distribution of the motifs to be found by MEME in the set of regulatory sequences corresponded to zero or one *per* sequence, the –*mod* argument of MEME was set to *zoops*.
- Background Markov model. In order to avoid the bias originated by the unbalance distribution of the nucleotides (i.e. low or high %GC) in the regulatory sequences, we build a Markov model file for each one of our six regulatory systems. The names of these files were specified using the –*bfile* argument of MEME.
- Alphabet of the sequences. The –*dna* argument of MEME was used to specify the nature of the nucleotide sequences used in our study.

### Mapping the significant motifs onto a reference sequence

To identify the relative positions of the different motifs identified in the previous steps of our protocol, every motif was mapped to a reference intergenic region of a model organism. In our case, we selected *E. coli* K12 because it is one of the best-characterized organisms among the Gammaproteobacteria. As a result of this mapping step, a PProCoM was obtained.

### Integration of the mapped motifs with biological knowledge of the regulatory system and construction of dynamic models of the regulatory system

To properly interpret the results obtained in the previous steps represented as a PProCoM, the molecular characteristics of the TF in the study were considered. The characteristics of the TFs belonging to the LysR-type family are listed in the Background section and include the following properties: the TF-TG divergent transcriptional orientations, the tandem arrangement of TFBSs, the inverted repeat symmetry and length of the TFs, the cooperative binding of the TFs in the presence of a specific inducer, the relative degrees of sequence conservation (i.e., binding affinities) of the TFBSs and their positions with respect to their promoters, and the spaces between the TFBSs that determine their relative orientations in terms of helix-turns.

## Additional file

Additional file 1: List of the 150 organisms whose genomic sequences were used in our study. (XLSX 69 kb)

## References

[1] Molina N, van Nimwegen E (2009). Scaling laws in functional genome content across prokaryotic clades and lifestyles. Trends Genet..

[2] Martínez-Antonio A, Collado-Vides J (2003). Identifying global regulators in transcriptional regulatory networks in bacteria. Curr Opin Microbiol..

[3] Pérez-Rueda E, Collado-Vides J (2000). The repertoire of DNA-binding transcriptional regulators in Escherichia coli K-12. Nucleic Acids Res..

[4] Huerta AM, Salgado H, Thieffry D, Collado-Vides J (1998). RegulonDB: a database on transcriptional regulation in Escherichia coli. Nucleic Acids Res..

[5] Tagle DA, Koop BF, Goodman M, Slightom JL, Hess DL, Jones RT (1988). Embryonic epsilon and gamma globin genes of a prosimian primate (galago crassicaudatus). Nucleotide and amino acid sequences, developmental regulation and phylogenetic footprints. J Mol Biol.

[6] Tan K, Moreno-Hagelsieb G, Collado-Vides J, Stormo GD (2001). A comparative genomics approach to prediction of new members of regulons. Genome Res..

[7] Tan K, McCue LA, Stormo GD (2005). Making connections between novel transcription factors and their DNA motifs. Genome Res..

[8] Janky R, van Helden J (2008). Evaluation of phylogenetic footprint discovery for predicting bacterial cis-regulatory elements and revealing their evolution. BMC Bioinformatics..

[9] Salgado H, Peralta-Gil M, Gama-Castro S, Santos-Zavaleta A, Muniz-Rascado L, Garcia-Sotelo JS (2013). RegulonDB v8.0: omics data sets, evolutionary conservation, regulatory phrases, cross-validated gold standards and more. Nucleic Acids Research.

[10] Keseler IM, Mackie A, Peralta-Gil M, Santos-Zavaleta A, Gama-Castro S, Bonavides-Martínez C (2013). EcoCyc: fusing model organism databases with systems biology. Nucleic Acids Res..

[11] Novichkov PS, Brettin TS, Novichkova ES, Dehal PS, Arkin AP, Dubchak I (2012). RegPrecise web services interface: programmatic access to the transcriptional regulatory interactions in bacteria reconstructed by comparative genomics. Nucleic Acids Res..

[12] Grote A, Klein J, Retter I, Haddad I, Behling S, Bunk B (2009). PRODORIC (release 2009): a database and tool platform for the analysis of gene regulation in prokaryotes. Nucleic Acids Res.

[13] Pérez AG, Angarica VE, Vasconcelos AT, Collado-Vides J (2007). Tractor_DB (version 2.0): a database of regulatory interactions in gamma-proteobacterial genomes. Nucleic Acids Res.

[14] Oberto J (2010). FITBAR: a web tool for the robust prediction of prokaryotic regulons. BMC Bioinformatics..

[15] Medina-Rivera A, Abreu-Goodger C, Thomas-Chollier M, Salgado H, Collado-Vides J, Van Helden J (2011). Theoretical and empirical quality assessment of transcription factor-binding motifs. Nucleic Acids Res..

[16] Schell MA (1993). Molecular biology of the LysR family of transcriptional regulators. Annu Rev Microbiol..

[17] Wilson RL, Steiert PS, Stauffer GV (1993). Positive regulation of the Escherichia coli glycine cleavage enzyme system. J Bacteriol..

[18] Wilson RL, Urbanowski ML, Stauffer GV (1995). DNA binding sites of the LysR-type regulator GcvA in the gcv and gcvA control regions of Escherichia coli. J Bacteriol..

[19] Urbanowski ML, Stauffer LT, Stauffer GV (2000). The gcvB gene encodes a small untranslated RNA involved in expression of the dipeptide and oligopeptide transport systems in Escherichia coli. Mol Microbiol..

[20] Stauffer LT, Stauffer GV (2005). GcvA interacts with both the alpha and sigma subunits of RNA polymerase to activate the Escherichia coli gcvB gene and the gcvTHP operon. FEMS Microbiol Lett..

[21] Jourdan AD, Stauffer GV (1999). Genetic analysis of the GcvA binding site in the gcvA control region. Microbiol..

[22] Wonderling LD, Urbanowski ML, Stauffer GV (2000). GcvA binding site 1 in the gcvTHP promoter of Escherichia coli is required for GcvA-mediated repression but not for GcvA-mediated activation. Microbiology..

[23] Cai XY, Maxon ME, Redfield B, Glass R, Brot N, Weissbach H (1989). Methionine synthesis in Escherichia coli: effect of the MetR protein on metE and metH expression. Proc Natl Acad Sci USA.

[24] Weissbach H, Brot N (1991). Regulation of methionine synthesis in Escherichia coli. Mol Microbiol..

[25] Flatley J, Barrett J, Pullan ST, Hughes MN, Green J, Poole RK (2005). Transcriptional responses of Escherichia coli to S-nitrosoglutathione under defined chemostat conditions reveal major changes in methionine biosynthesis. J Biol Chem..

[26] Membrillo-Hernández J, Coopamah MD, Channa A, Hughes MN, Poole RK (1998). A novel mechanism for upregulation of the Escherichia coli K-12 hmp (flavohaemoglobin) gene by the “NO releaser”, S-nitrosoglutathione: nitrosation of homocysteine and modulation of MetR binding to the glyA-hmp intergenic region. Mol Microbiol..

[27] Maxon ME, Redfield B, Cai XY, Shoeman R, Fujita K, Fisher W (1989). Regulation of methionine synthesis in Escherichia coli: effect of the MetR protein on the expression of the metE and metR genes. Proc Natl Acad Sci U S A..

[28] Jafri S, Urbanowski ML, Stauffer GV (1995). A mutation in the rpoA gene encoding the alpha subunit of RNA polymerase that affects metE-metR transcription in Escherichia coli. J Bacteriol..

[29] Wu WF, Urbanowski ML, Stauffer GV (1995). Characterization of a second MetR-binding site in the metE metR regulatory region of salmonella typhimurium. J Bacteriol..

[30] Lorenz E, Stauffer GV (1995). Characterization of the MetR binding sites for the glyA gene of Escherichia coli. J Bacteriol..

[31] Lorenz E, Stauffer GV (1996). Cooperative MetR binding in the Escherichia coli glyA control region. FEMS Microbiol Lett..

[32] Harari O, del Val C, Romero-Zaliz R, Shin D, Huang H, Groisman EA (2009). Identifying promoter features of co-regulated genes with similar network motifs. BMC Bioinformatics.

[33] Collado-Vides J, Salgado H, Morett E, Gama-Castro S, Jiménez-Jacinto V, Martínez-Flores I (2009). Bioinformatics resources for the study of gene regulation in bacteria. J Bacteriol..

[34] Urbanowski ML, Stauffer GV (1989). Genetic and biochemical analysis of the MetR activator-binding site in the metE metR control region of Salmonella typhimurium. J Bacteriol.

[35] Anjem A, Varghese S, Imlay JA (2009). Manganese import is a key element of the OxyR response to hydrogen peroxide in Escherichia coli. Mol Microbiol..

[36] Storz G, Tartaglia LA, Ames BN (1990). The OxyR regulon. Antonie van Leeuwenhoek.

[37] Zheng M, Wang X, Templeton LJ, Smulski DR, LaRossa RA, Storz G (2001). DNA microarray-mediated transcriptional profiling of the Escherichia coli response to hydrogen peroxide. J Bacteriol..

[38] Mongkolsuk S, Helmann JD (2002). Regulation of inducible peroxide stress responses. Mol Microbiol..

[39] Zheng M, Aslund F, Storz G (1998). Activation of the OxyR transcription factor by reversible disulfide bond formation. Science..

[40] Toledano MB, Kullik I, Trinh F, Baird PT, Schneider TD, Storz G (1994). Redox-dependent shift of OxyR-DNA contacts along an extended DNA-binding site: A mechanism for differential promoter selection. Cell..

[41] Tartaglia LA, Gimeno CJ, Storz G, Ames BN (1992). Multidegenerate DNA recognition by the OxyR transcriptional regulator. J Biol Chem..

[42] Rhee KY, Senear DF, Hatfield GW (1998). Activation of gene expression by a ligand-induced conformational change of a protein-DNA complex. J Biol Chem..

[43] Wek RC, Hatfield GW (1988). Transcriptional activation at adjacent operators in the divergent-overlapping ilvY and ilvC promoters of Escherichia coli. J Mol Biol..

[44] Sung YC, Fuchs JA (1988). Characterization of the cyn operon in Escherichia coli K12. J Biol Chem..

[45] Lamblin AF, Fuchs JA (1993). Expression and purification of the CynR regulatory gene product: CynR is a DNA-binding protein. J Bacteriol..

[46] Lamblin AF, Fuchs JA (1994). Functional analysis of the Escherichia coli K-12 cyn operon transcriptional regulation. J Bacteriol..

[47] Stragier P, Richaud F, Borne F, Patte JC (1983). Regulation of diaminopimelate decarboxylase synthesis in Escherichia coli. I. Identification of a lysR gene encoding an activator of the lysA gene. J Mol Biol.

[48] Stragier P, Danos O, Patte JC (1983). Regulation of diaminopimelate decarboxylase synthesis in Escherichia coli. II. Nucleotide sequence of the lysA gene and its regulatory region. J Mol Biol.

[49] Stragier P, Patte JC (1983). Regulation of diaminopimelate decarboxylase synthesis in Escherichia coli. III. Nucleotide sequence and regulation of the lysR gene. J Mol Biol.

[50] Huerta AM, Collado-Vides J (2003). Sigma70 promoters in Escherichia coli: specific transcription in dense regions of overlapping promoter-like signals. J Mol Biol..

[51] Goethals K, Van Montagu M, Holsters M (1992). Conserved motifs in a divergent nod box of Azorhizobium caulinodans ORS571 reveal a common structure in promoters regulated by LysR-type proteins. Proc Natl Acad Sci U S A..

[52] Maddocks SE, Oyston PC (2008). Structure and function of the LysR-type transcriptional regulator (LTTR) family proteins. Microbiology..

[53] Parsek MR, Ye RW, Pun P, Chakrabarty AM (1994). Critical nucleotides in the interaction of a LysR-type regulator with its target promoter region. catBC promoter activation by CatR. J Biol Chem.

[54] Wang L, Winans SC (1995). The sixty nucleotide OccR operator contains a subsite essential and sufficient for OccR binding and a second subsite required for ligand-responsive DNA bending. J Mol Biol..

[55] Akakura R, Winans SC (2002). Mutations in the occQ operator that decrease OccR-induced DNA bending do not cause constitutive promoter activity. J Biol Chem..

[56] MacLean AM, Haerty W, Golding GB, Finan TM (2011). The LysR-type PcaQ protein regulates expression of a protocatechuate-inducible ABC-type transport system in Sinorhizobium meliloti. Microbiology..

[57] Wang L, Winans SC (1995). High angle and ligand-induced low angle DNA bends incited by OccR lie in the same plane with OccR bound to the interior angle. J Mol Biol..

[58] Parsek MR, McFall SM, Shinabarger DL, Chakrabarty AM (1994). Interaction of two LysR-type regulatory proteins CatR and ClcR with heterologous promoters: functional and evolutionary implications. Proc Natl Acad Sci U S A.

[59] MacLean AM, Anstey MI, Finan TM (2008). Binding site determinants for the LysR-type transcriptional regulator PcaQ in the legume endosymbiont Sinorhizobium meliloti. J Bacteriol.

[60] Hryniewicz MM, Kredich NM (1994). Stoichiometry of binding of CysB to the cysJIH, cysK, and cysP promoter regions of salmonella typhimurium. J Bacteriol..

[61] McFall SM, Klem TJ, Fujita N, Ishihama A, Chakrabarty AM (1997). DNase I footprinting, DNA bending and in vitro transcription analyses of ClcR and CatR interactions with the clcABD promoter: evidence of a conserved transcriptional activation mechanism. Mol Microbiol.

[62] Gallegos MT, Schleif R, Bairoch A, Hofmann K, Ramos JL (1997). Arac/XylS family of transcriptional regulators. Microbiol Mol Biol Rev.

[63] Felsenstein J (1981). Evolutionary trees from DNA sequences: a maximum likelihood approach. J Mol Evol..

[64] Ciccarelli F, Doerks T, Von MC (2006). Toward automatic reconstruction of a highly resolved tree of life. Science..

[65] Zhang M, Leong HW (2010). Bidirectional best hit r-window gene clusters. BMC Bioinformatics.

[66] Altschul SF, Madden TL, Schäffer AA, Zhang J, Zhang Z, Miller W, Lipman DJ (1997). Gapped BLAST and PSI-BLAST: a new generation of protein database search programs. Nucleic Acids Res..

[67] Bailey TL, Boden M, Buske FA, Frith M, Grant CE, Clementi L (2009). MEME suite: tools for motif discovery and searching. Nucleic Acids Res..

